# Obesity and epithelial ovarian cancer survival: a systematic review and meta-analysis

**DOI:** 10.1186/1757-2215-7-41

**Published:** 2014-04-22

**Authors:** Hyo Sook Bae, Hyun Jung Kim, Jin Hwa Hong, Jae Kwan Lee, Nak Woo Lee, Jae Yun Song

**Affiliations:** 1 Departments of Obstetrics and Gynecology, Korea University College of Medicine, Seoul, Korea; 2 Department of Preventive Medicine, Korea University College of Medicine, Seoul, Korea

**Keywords:** Ovarian Neoplasms, Obesity, Body mass index, Survival

## Abstract

**Background:**

Studies on the association between obesity and ovarian cancer survival have had conflicting results. We reviewed and quantitatively summarized the existing evidence, exploring potentially important sources of variability, such as the timing of body mass index (BMI) assessment, BMI cut points, references used in multivariate analysis, and ovarian cancer stage.

**Methods:**

Eligible studies were searched using MEDLINE (PubMed), EMBASE, and Cochrane Central Register of Controlled Trials, relevant bibliographies were manually reviewed for additional studies. Adjusted hazard ratios (HRs) from individual studies were pooled using a random effects model.

**Results:**

17 cohort studies of 929 screened articles were included in the final analysis. Obesity in early adulthood and obesity 5 years before ovarian cancer diagnosis were associated with poor patient survival (early adulthood: pooled HR 1.67; 95% CI 1.29-2.16; 5 years prediagnosis: pooled HR 1.35; 95% CI 1.03-1.76). However, the results for obesity at diagnosis depended on whether BMI was analyzed as a categorical or continuous variable. Analysis of obesity with BMI as a categorical variable did not affect ovarian cancer prognosis (pooled HR 1.07; 95% CI 0.95-1.21); obesity with BMI as a continuous variable showed slightly poorer survival with each incremental increase in BMI (pooled HR 1.02; 95% CI 1.01-1.04).

**Conclusions:**

Obesity 5 years before ovarian cancer diagnosis and obesity at a young age were associated with poor prognosis. The association between obesity at diagnosis and survival of ovarian cancer patients still remains equivocal. BMI at diagnosis cannot be a prognostic factor for the survival of ovarian cancer patients. Further well-designed studies are needed to elucidate the variety effect of obesity on the survival of ovarian cancer patients.

## Background

Ovarian cancer is highly fatal gynecological cancer. It is the fifth leading cause of cancer mortality in women with 14,030 deaths each year in the United States [1]. Obesity is a rising health problem worldwide with an increasing population of obese people and direct links between obesity and multiple morbidities. Among gynecological cancers, hormone-related cancers such as endometrial cancer and breast cancer are related to obesity [2]. Some epidemiological studies report that obesity is related to ovarian cancer incidence [3].

However, results on the relationship between epithelial ovarian cancer and obesity are conflicting. Recently, Pavelka et al. reported that obesity affects ovarian cancer mortality by influencing tumor biology [4]. However, many studies report no significant change in survival according to body mass index (BMI) [5,6]. Most ovarian cancer patients require an operation and toxic chemotherapy that can negatively affect their health. Furthermore, advanced ovarian cancer patients are frequently cachectic, with ascites that affects BMI but is not true body mass. Therefore, whether obesity has a true adverse effect on outcomes of ovarian cancer patients is unknown.

Two studies included a meta-analysis that reported the relationship between obesity and ovarian cancer survival. Protani et al., using 2007 data, reported that women with ovarian cancer who were obese appeared to have slightly worse survival than nonobese women [7]. However, the association was valid only for studies that included women with BMI ≥30. Yang et al., using 2010 data, reported that obesity in early adulthood is related to higher mortality among patients with ovarian cancer [8]. However, only studies using BMI as a categorical variable were included.

In this study, we reviewed the current literature for an association between obesity and survival of women with ovarian cancer. We conducted a comprehensive meta-analysis to determine the impact of obesity as a risk factor on the prognosis of ovarian cancer, analyzing the timing of BMI assessment and the methods used to analyze BMI as a variable.

## Methods

### Search strategy

This systematic review and meta-analysis were conducted according to the Meta-analysis of Observational Studies in Epidemiology (MOOSE) guidelines [9]. A systematic search to June 2013 of MEDLINE (PubMed), EMBASE and Cochrane Central Register of Controlled Trials (CENTRAL) in the Cochrane Library was conducted to identify eligible studies on the association between obesity and survival in women with ovarian cancer. Keywords were: (obesity OR overweight OR “body mass index” OR “body size” OR “body weight”) AND (“ovarian cancer” OR “ovarian neoplasm” OR “ovarian malignancy”) AND (survival analysis OR survival rate OR survival OR death OR mortality OR morbidity OR prognosis). The reference lists of all eligible articles and reviews were also manually scanned to identify additional studies for inclusion.

### Study selection and data extraction

Eligibility criteria for inclusion in the systematic review were (1) original data examining the association between obesity and survival in a cohort of patients with ovarian cancer, and (2) outcome measures reported as adjusted hazard ratios (HRs). Two authors (H-S.B. and J-Y.S.) independently evaluated the eligibility of all studies retrieved from the databases. For all eligible studies, information was extracted on study design, country, year of diagnosis, number of years of follow up, participant ages, tumor stage, BMI definitions and categories, timing of BMI measurement, median patient survival, effect estimates, and variables included in analysis adjustment.

### Statistical analysis

HR estimates were pooled using random-effects analysis using the method of DerSimonian and Laird, and heterogeneity across studies was assessed using the I^2^ statistic from Higgins and Thompson, which measures the percentage of total variation across studies [10,11]. Pooled HR with 95% CIs, were determined using adjusted HRs and CIs reported in the articles or obtained from the authors. When several HR values were given in an article, the value adjusted for most confounders was used. Subgroup analyses were by BMI category, BMI measurement timing, and BMI group, which was the reference for regression analysis. In four studies, the effect of obesity on ovarian cancer survival was reported with BMI as a continuous variable; we pooled those studies separately [4,12-14]. Publication bias was assessed by examining funnel plot asymmetry. We assessed the quality of included studies using the Newcastle-Ottawa scale [15]. Quality scores were calculated based on three major components: (1) selection of groups to study, (2) comparability, and (3) assessment of outcome or exposure. The quality scores of included studies were similar, ranging from 5 to 8 (Additional files 1 and 2) with a maximum score of 9, representing the highest methodological quality. Sensitivity analyses determined differences in study design, sample size, study quality grade, and diagnostic criteria for obesity. Statistical analyses were conducted using Review Manager, ver5.2 (Nordic Cochrane Center, Copenhagen, Denmark).

## Results

### Identification of relevant studies

**Figure 1:**
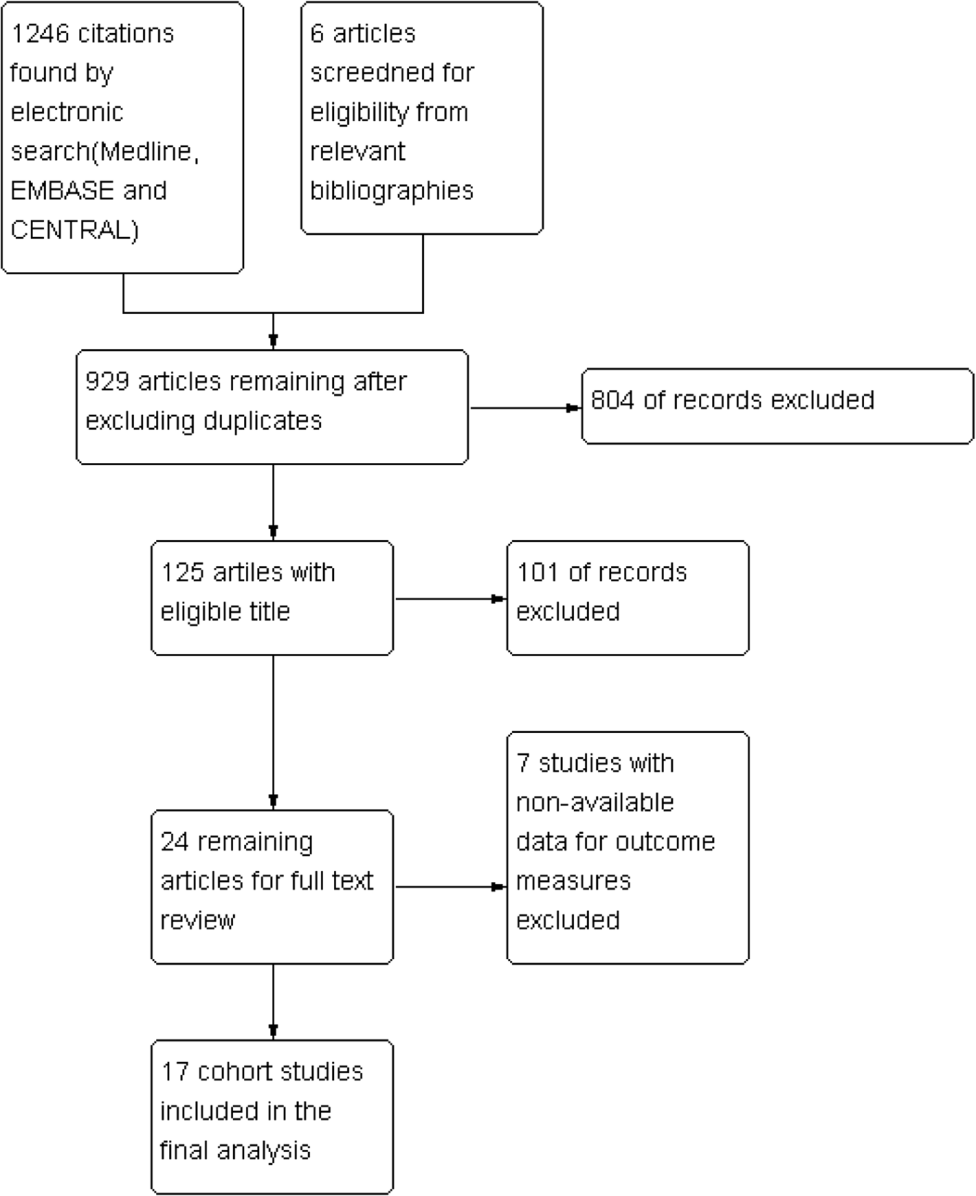
Flow diagram.

A total of 929 citations were found by searching MEDLINE, EMBASE and CENTRAL; 804 articles were excluded during title review and 101 articles were excluded during abstract review. Thus, 24 articles underwent full text review, and 17 cohort studies were included in the final meta-analysis (Figure 1, Table 1). General characteristics of the included studies are in Table 1.

**Table 1:** Characteristics of studies

**Study**	**Country**	**N**	**Years of diagnosis**	**Follow-up, yr**	**Stage**	**Age, yr**	**Timing of BMI measurement**	**BMI category**	**Adjustment variables**
Min Zhang et al.	China	207	1999-2000	Minimum, 3	all stages		1. at diagnosis	BMI < 20.0	Age, stage, grade, ascites, residual lesions, chemotherapy, total energy intake, menopausal status
						Mean alive, 46.7	2. 5 year ago	20.0 ≤ BMI ≤ 22.4	
						Mean dead, 51.6	3. at age 21 y	22.5 ≤ BMI ≤ 24.9	
								25.0 ≤ BMI	
Joanne Kotsopoulos et al.	Canada	1423	1995-2004	mean 7.4	all stages	under, 50.75	5 years prior to diagnosis	BMI < 18.5	Age at diagnosis, BRCA mutation status, stage and histologic subtype
				range, 0.59-15.72					
						normal, 56.23		18.5 ≤ BMI ≤ 25	
						overweight, 57.84		25 ≤ BMI ≤ 30	
						obese, 57.78		30 ≤ BMI	
Yang Zhou et al.	USA	388	1998-2003	all: 9.28 ± 8.68	all stages	Median, 58.6	1. during 20s	BMI < 25	Age, stage, histology, education, oral contraceptive use, menopausal status and HRT use, parity, age at first birth, family history of ovarian cancer, time from ovarian cancer diagnosis to study enrollment
								BMI ≥ 25	
				BMI < 25: 8.67 ± 7.96			2. 5 years before diagnosis	BMI < 25	
								BMI ≥ 25	
				BMI ≥ 25: 9.95 ± 9.38			3. 9 months post-chemotherapy		
Ling Yang et al.	National wide (UK, Sweden, Italy, Norway, Finland)	635	1993-1995	Range, 50-74	all stages	50-74	1. Age 18	BMI < 18.5	Age at diagnosis, FIGO stage and WHO grade of differentiation
								18.5 ≤ BMI ≤ 25	
							2. 1 year prior to ovarian cancer diagnosis	25 ≤ BMI ≤ 30	
								30 ≤ BMI	
I. SKI’RNISDO’ TTIR et al.	Sweden	635	1975-2004	Mean, 6.8	I,II	Mean 60.1	at the start of the adjuvant therapy	BMI < 18.5	Stage, grade, histology
								18.5 ≤ BMI ≤ 25	
								25 ≤ BMI ≤ 30	
								30 ≤ BMI	
				Range, 1.6-17.8					
Anette Kjærbye-Thygesen et al.	Denmark	295	1994-1999	Median, 7.3	III	Range, 35-79	1. BMI age at 20-29y	BMI < 18.5	Age, radicality of surgery, histology, platinum-based chemotherapy, smoking status, continuous BMI 5 years before diagnosis
								18.5 ≤ BMI ≤ 24.9	
				Range, 5.4-9.5			2. BMI 5years before diagnosis	25.0 ≤ BMI	
Crystal P. Tyler,	USA	425	1980-1982	Median, 9.7	all stages	20-54	1. adult BMI(within 6 months of diagnosis)	lowest quartile (<20.7) the second (20.8–22.5) third (22.6–24.9) fourth (≥25.0) quartiles	Age at diagnosis, stage at diagnosis, histologic type, oral contraceptive use, parity, menopausal status, presence of any other chronic conditions including diabetes, high blood pressure, chronic kidney disease, gallbladder disease, myocardial infarction, heart disease, high cholesterol, paralysis, or stroke
							2. BMI at age 18,		
							3. weight change from age 18 to adult		
Kirsten B. Moysich et al.	USA	395	1982-1998	NA (≥9)	all stages	mean(SD)	self-reported	BMI < 18.5	age at diagnosis, FIGO stage
						alive: 47.5(14.1)	1. current height and weight	18.5 ≤ BMI ≤ 25	
						dead: 58.3(12.3)	2. weight before dx	25 ≤ BMI ≤ 30	
								30 < BMI	
INGIRIDUR SKÍRNISDÓTTIR & BENGT SORBE	Sweden	446	1994-2003	Mean, 3.9	all stages	Mean, 62.5	at the start of the	BMI ≤25	Age, stage, histology
				Range, 0-12.3		range, 25-91	adjuvant therapy	BMI > 25	
James C. Pavelka et al.	USA	149	1996-2003	Not stated	III-IV	Range, 18-79	first postoperative visit	BMI < 18.5	nil
								18.5 ≤ BMI ≤ 25	
								25 ≤ BMI ≤ 30	
								30 < BMI	
Schlumbrecht, M.	USA	127	2002-2007	mean, 3.1 (0.3-7.2)	not stated	not stated	not stated	BMI < 18.5	not stated
								18.5 ≤ BMI ≤ 25	
								25 ≤ BMI ≤ 30	
								30 < BMI	
Dolecek, T.A. et al.	USA	341	1994-1998	Not stated	I-IV	Range, 18-74	Self-reported BMI at diagnosis	BMI < 18.5	Age, race, stage, grade, residual lesions, smoking status, oral contraceptive use, parity
								18.5 ≤ BMI ≤ 25	
								25 ≤ BMI ≤ 30	
								30 ≤ BMI	
Fotopoulou, C. et al.	Germany	306	2000-2010	11.7 months (0.1-62.9)	I-IV	58(18–92)	Not stated	BMI < 25	Residual tumor, grade, positive lymph node status, age, FIGO stage, Ascites, IMO level 2/3 involvement, nonserous histology, distant metastasis
								BMI ≥ 25	
Lamkin, D.M. et al.	USA	74	2001-2005	Not stated	I-IV	62(33–87)	Not stated	BMI as continuous variable	None (univariate analysis)
Nagle, C.M. et al.	Australia	609	1990-1993	Median 7.3 yrs (5–8.3)	I-IV	18-79	Prior to illness	BMI <22.2	FIGO stage, age, grade, total energy intake (Kilocalories), BMI, residual, ascites, smoking status, parity and length of OCP use
								22.2-25.8	
								BMI > 25.8	
Schlumbrecht, M.P. et al.	USA	194	1977-2009	Median f/u 60.9 months (1–383)	I-IV	44.9(14–79)	8 wks after primary surgical intervention	BMI <25	Stage, Taxane, Current alcohol use, year of diagnosis, current smoker, age at diagnosis, hormone tx after adjuvant ctx
								25 ≤ BMI < 30	
								30 ≤ BMI < 35	
								35 ≤ BMI	
Schildkraut, J.M. et al.	USA	197	1980-1982	Not stated	I-IV	20-54	At diagnosis	BMI > 27.9	None (univariate analysis)

IMO, Intraoperative Mapping of Ovarian Cancer.

## Meta-analysis

### Association between obesity before diagnosis and ovarian cancer patient survival

**Figure 2:**
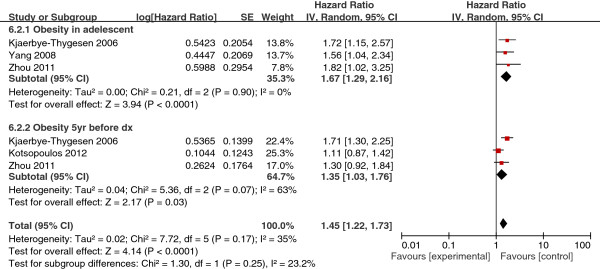
Obesity before diagnosis. (Note: BMI ≥ 25–30).

Four studies reported HRs related to obesity before diagnosis. Three studies that included BMI in adolescence in analyses were pooled, yielding a summary HR estimate of 1.67 (95% CI, 1.29-2.16). Three studies analyzing BMI 5 years before diagnosis showed similar results (HR, 1.35; 95% CI, 1.03-1.76) (Figure 2).

### Association between obesity at diagnosis and ovarian cancer patient survival

**Figure 3:**
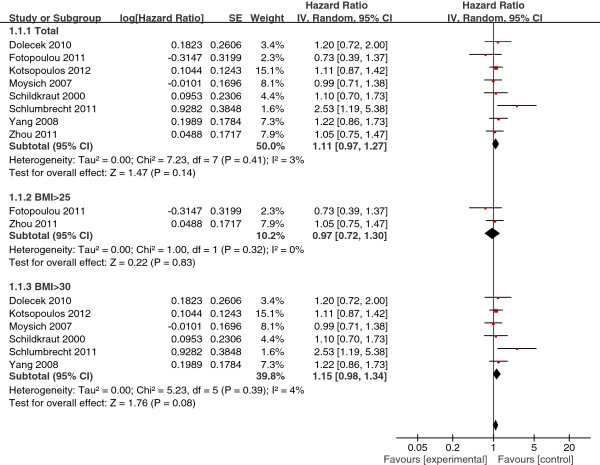
Obesity at diagnosis (normal weight as reference).

**Figure 4:**
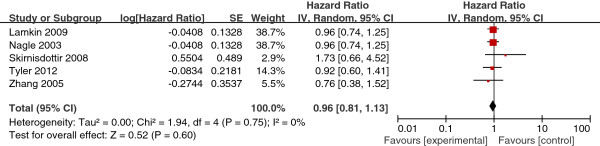
Obesity at diagnosis (low-weight as reference).

**Figure 5:**
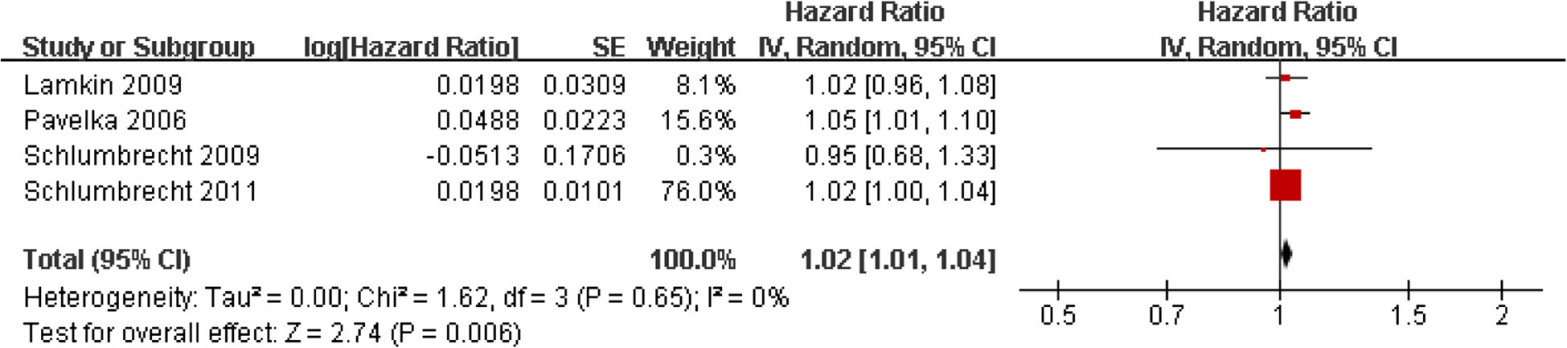
BMI as continuous variable.

The selected studies that included BMI at diagnosis in analyses had substantial interstudy heterogeneity in the BMI cutoff used to define obesity, the reference group for analysis; and the form of the BMI variable, continuous or categorical. Eight studies used data on obesity at diagnosis and survival of ovarian cancer patients using a normal weight group as the reference group. Pooling the data from the eight studies resulted in a summary HR estimate of 1.11 (95% CI, 0.97-1.27). Subgroup analysis of two studies with BMI ≥ 25 as the cutoff value for obesity yielded a summary HR estimate of 0.97 (95% CI, 0.72-1.30). Analysis of six studies with BMI >30 as obese yielded a summary HR estimate of 1.15 (95% CI, 0.98-1.38) (Figure 3). Five studies used a low-weight group as a reference group in analyses. Pooling these five cohort studies yielded a summary HR of 0.96 (95% CI, 0.81-1.13) (Figure 4). Four studies analyzed BMI as a continuous variable. The pooled summary HR of these studies was 1.02 (95% CI, 1.01-1.04) per incremental BMI unit (Figure 5).

## Discussion

For almost every health condition, obese patients show poorer outcome than non-obese patients because obese patients generally have more comorbidities such as hypertension, diabetes, and MI [16,17]. Nevertheless, no conclusive relationship has been established between obesity and ovarian cancer patient survival.

In this meta-analysis, we investigated the association between obesity before diagnosis (at 5 years before diagnosis and at a young age) and ovarian cancer patient survival. The association between obesity at the time of diagnosis and ovarian cancer survival was weaker than the association between BMI evaluated before diagnosis and ovarian cancer survival.

This study is the largest meta-analysis using current data on the association between obesity and ovarian cancer survival. Although two meta-analyses were previously published, our results are noteworthy because some of our results differ from previous studies and some are more reliable because of strictly structured subgroup analysis.

Obesity at a young age and before ovarian cancer diagnosis appeared to be related to poor cancer outcome. Yang et al. reported similar results, showing a possible relationship between obesity in early adulthood and higher mortality [8]. However, two previous meta-analyses reported different results about the association between BMI at diagnosis and ovarian cancer survival. Protani et al. reported a pooled HR of 1.13 (95% CI; 0.81-1.57) for BMI at diagnosis and ovarian cancer survival with a slightly stronger association in studies that defined only women with BMI ≥ 30 as obese. Protani et al. concluded that women with ovarian cancer who are obese have slightly worse survival outcomes than nonobese women [7]. Our results are similar to Yang et al., which reported no significant relationship between prognosis and obesity at diagnosis [8]. Our study includes more cohort studies than Yang et al.

Interestingly, our study showed two different results in the association between BMI at diagnosis and ovarian cancer survival. Although only four studies were included in our meta-analysis, BMI as a continuous variable was related to poor ovarian cancer outcome (Figure 5). The HR of 1.02 per incremental BMI unit was not clinically insignificant because this HR estimate suggest a 10% increase in mortality with 5-unit increase in BMI.

We support the hypothesis that obesity has adverse effects on the mortality in the general population [16,17]. In our study, the relationship between obesity before diagnosis and ovarian cancer patient mortality was similar to results on the general population. We suggest that the weak relationship between obesity at diagnosis and ovarian cancer survival was due to some factors of ovarian cancer patients that interfered with or weakened the potential adverse effects of obesity on health.

We propose several reasons for the weak relationship between obesity at diagnosis and ovarian cancer survival. First, BMI is not an appropriate measure for evaluating the degree of obesity in ovarian cancer patients because they often have ascites or cachexia [18,19]. Second, obese or overweight patients with ovarian cancer might endure toxic chemotherapy better than nonobese patients. Third, an unknown action of obesity might improve ovarian cancer outcomes.

Although we tried to overcome inference by confounding factors, primary observational cohort studies have inherent limitations. Additional well-designed epidemiological and laboratory studies could reveal the true effects of obesity on ovarian cancer survival.

## Conclusions

The results of our meta-analysis suggested that obesity before cancer diagnosis was associated with poor ovarian cancer patient survival. However, the adverse effect of obesity on ovarian cancer survival was still equivocal for BMI measured at the time of diagnosis. BMI at diagnosis cannot be a prognostic factor for the survival of ovarian cancer patients. Further well-designed studies are needed to elucidate the variety effect of obesity on the survival of ovarian cancer patients.

## Competing interests

The authors declare that they have no competing interests.

## Authors’ contributions

J-YS and H-SB were the gynecologists who designed this study and extracted data used in the final meta-analysis. H-JK was the statistician who confirmed the analysis of this study. All authors read and approved the final manuscript.

## Supplementary Material

Additional file 1: Table S1 Risk of Bias. Click here for file

Additional file 2: Figure S1 Funnel plots of the meta-analyses. Click here for file
