# Supplementary material for: Obesity and epithelial ovarian cancer survival: a systematic review and meta-analysis
Source: J Ovarian Res. 2014 Apr 22;7:41. doi: 10.1186/1757-2215-7-41 (PMC4022349; doi:10.1186/1757-2215-7-41)
Supplement: Additional file 1: Table S1 — Risk of Bias. [file 1757-2215-7-41-S1.doc]

Additional file 1: Table S1. Risk of Bias

| Study | Selection | Comparability | Outcome |
| --- | --- | --- | --- |
| Zhang 2005 | ☆☆☆ | ☆☆ | ☆☆☆ |
| Kotsopoulos 2012 | ☆☆ | ☆☆ | ☆☆ |
| Zhou 2011 | ☆☆☆ | ☆ | ☆☆☆ |
| Yang 2008 | ☆☆☆ | ☆☆ | ☆☆☆ |
| SKIRNISDOTTIR 2008 | ☆☆ | ☆ | ☆☆☆ |
| Kjaerbye-Thygesen 2006 | ☆☆ | ☆☆ | ☆☆ |
| Tyler 2012 | ☆☆ | ☆☆ | ☆☆ |
| Moysich 2007 | ☆☆☆ | ☆ | ☆ |
| SKIRNISDOTTIR 2010 | ☆☆☆ | ☆☆ | ☆☆ |
| Pavelka 2006 | ☆☆☆ | ☆☆ | ☆☆☆ |
| Schlumbrecht 2009. | ☆☆ | ☆ | ☆ |
| Dolecek 2010 | ☆☆☆ | ☆☆ | ☆☆ |
| Fotopoulou 2011 | ☆☆☆ | ☆☆ | ☆☆ |
| Lamkin 2009 | ☆☆ | ☆ | ☆ |
| Nagle 2003 | ☆☆ | ☆☆ | ☆☆☆ |
| Schlumbrecht 2011 | ☆ | ☆☆ | ☆☆☆ |
| Schildkraut 2000 | ☆☆☆ | ☆ | ☆ |
