# Supplementary material for: Obesity and epithelial ovarian cancer survival: a systematic review and meta-analysis
Source: J Ovarian Res. 2014 Apr 22;7:41. doi: 10.1186/1757-2215-7-41 (PMC4022349; doi:10.1186/1757-2215-7-41)

**Additional file 2: Figure S1 Funnel plots of the meta-analyses.**

**(Manuscript Figure 2.) Obesity before diagnosis. (Note: BMI ≥ 25-30)**


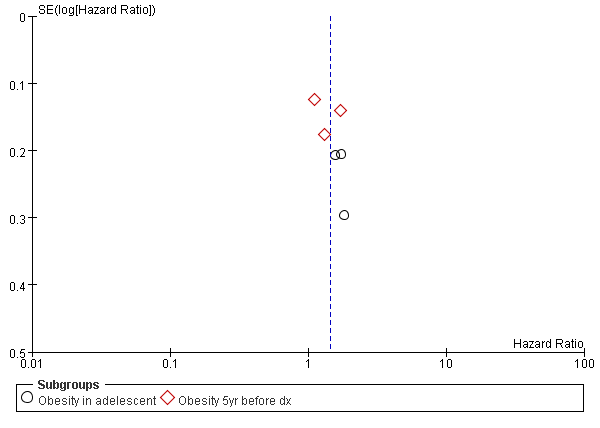


**(Manuscript Figure 3.) Obesity at diagnosis (normal weight as reference)**


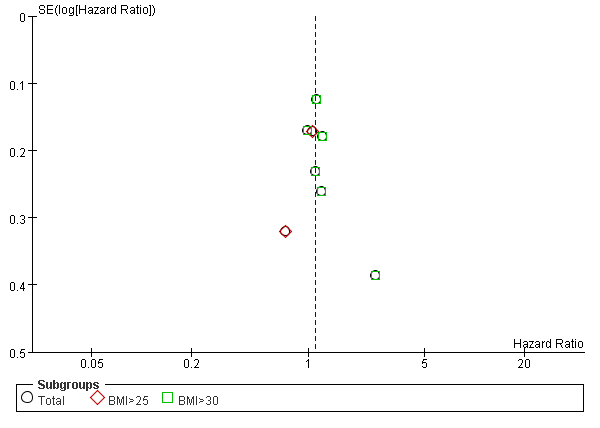


**(Manucript Figure 4.) Obesity at diagnosis (low-weight as reference)**


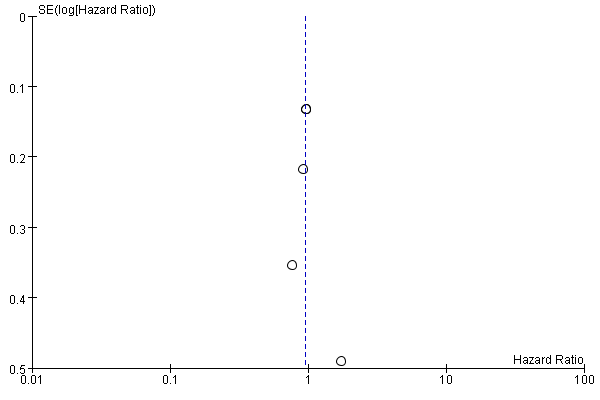


**(Manuscript Figure 5.) BMI as continuous variable**


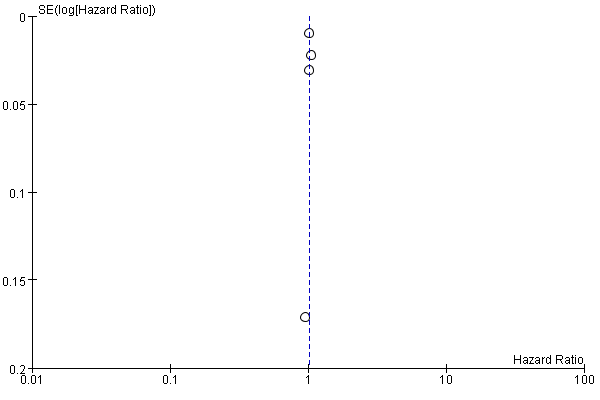

Supplement: Additional file 2: Figure S1 — Funnel plots of the meta-analyses. [file 1757-2215-7-41-S2.doc]
